# Notch Signaling Regulates Bile Duct Morphogenesis in Mice

**DOI:** 10.1371/journal.pone.0001851

**Published:** 2008-03-26

**Authors:** Julie Lozier, Brent McCright, Thomas Gridley

**Affiliations:** The Jackson Laboratory, Bar Harbor, Maine, United States of America; Centre de Regulacio Genomica, Spain

## Abstract

**Background:**

Alagille syndrome is a developmental disorder caused predominantly by mutations in the Jagged1 (*JAG1*) gene, which encodes a ligand for Notch family receptors. A characteristic feature of Alagille syndrome is intrahepatic bile duct paucity. We described previously that mice doubly heterozygous for *Jag1* and *Notch2* mutations are an excellent model for Alagille syndrome. However, our previous study did not establish whether bile duct paucity in *Jag1/Notch2* double heterozygous mice resulted from impaired differentiation of bile duct precursor cells, or from defects in bile duct morphogenesis.

**Methodology/Principal Findings:**

Here we characterize embryonic biliary tract formation in our previously described *Jag1/Notch2* double heterozygous Alagille syndrome model, and describe another mouse model of bile duct paucity resulting from liver-specific deletion of the *Notch2* gene.

**Conclusions/Significance:**

Our data support a model in which bile duct paucity in Notch pathway loss of function mutant mice results from defects in bile duct morphogenesis rather than cell fate specification.

## Introduction

The primary functional cells of the mammalian liver are the hepatocytes and the epithelial bile duct cells, or cholangiocytes (for recent reviews, see references [1]–[3]). During liver development, both hepatocytes and cholangiocytes differentiate from bipotential progenitor cells termed hepatoblasts [4], [5]. Hepatoblasts located in the liver parenchyma differentiate into hepatocytes, while hepatoblasts located at the interface of the portal mesenchyme (which surrounds the portal vein) and the liver parenchyma differentiate into the biliary epithelial cells. Initially, biliary epithelial cells form a continuous single cell layer termed the ductal plate (reviewed in [5]). The ductal plate subsequently undergoes morphogenesis and remodeling to generate the epithelial bile ducts. Defects in bile duct formation can lead to an impairment of bile duct flow (cholestasis), and result in a diverse group of both genetic and acquired biliary tract disorders termed cholangiopathies (reviewed in [6], [7]).

The Notch signaling pathway is an evolutionarily conserved intercellular signaling mechanism (reviewed in [8], [9]), and mutations in its components disrupt embryonic development in diverse organisms and cause inherited disease syndromes in humans. Mutations in the *JAG1* gene, which encodes a ligand for Notch family receptors, cause Alagille syndrome [10], [11]. Alagille syndrome (OMIM #118450) is a pleiotropic developmental disorder characterized by cholestasis and jaundice caused by intrahepatic bile duct paucity, congenital heart defects, vertebral defects, eye abnormalities, facial dysmorphism, and kidney abnormalities [12]–[14]. Alagille syndrome exhibits autosomal dominant inheritance, and analysis of the types of *JAG1* mutations in Alagille syndrome patients suggest *JAG1* haploinsufficiency as the primary cause of Alagille syndrome.

We have described previously a mouse model for Alagille syndrome [15]. Mice heterozygous for a *Jag1* null allele, which have the same genotype as Alagille syndrome patients, exhibited haploinsufficient eye defects but did not exhibit other phenotypic abnormalities characteristic for Alagille syndrome [16]. However, mice doubly heterozygous for a *Jag1* null allele and a *Notch2* hypomorphic allele exhibited most of the clinically relevant features of Alagille syndrome, including bile duct paucity [15]. Our previous studies of these mice concentrated on analysis of late embryonic and postnatal livers, and did not establish whether bile duct paucity in *Jag1/Notch2* double heterozygous mice was due to defects in differentiation of bile duct precursors from the bipotential hepatoblast, or defects in morphogenesis of the ductal plate.

A recent study of Hairy and enhancer of split 1 (*Hes1*)-null mice suggested that the role of Notch signaling during biliary development was in the control of biliary tract morphogenesis, rather than in a hepatocyte-cholangiocyte cell fate specification decision [17]. However, other genes encoding *Hes*-related bHLH proteins are also Notch targets, raising the possibility that *Hes1*-null mice may not reflect the full extent of the role played by the Notch signaling pathway during biliary development. In addition, since *Hes1*-null mice die perinatally from defects unrelated to the liver defects [18], morphogenesis and maturation of the intrahepatic biliary system cannot be followed during the early postnatal period when major biliary tract remodeling and maturation events take place [5].

In this paper, we characterize embryonic biliary tract formation in the previously described *Jag1/Notch2* double heterozygote mouse model of Alagille syndrome. We also describe another mouse model of bile duct paucity resulting from liver-specific deletion of the *Notch2* gene. Our data demonstrate a requirement for *Jag1/Notch2*-mediated signaling in bile duct formation in mice, and support a model in which bile duct paucity in Notch pathway loss of function mutant mice results from defects in bile duct morphogenesis rather than cell fate specification.

## Results

### Analysis of bile duct morphogenesis during embryogenesis in *Jag1^dDSL^/+ Notch2^del1^/+* double heterozygous mice

**Figure 1:**
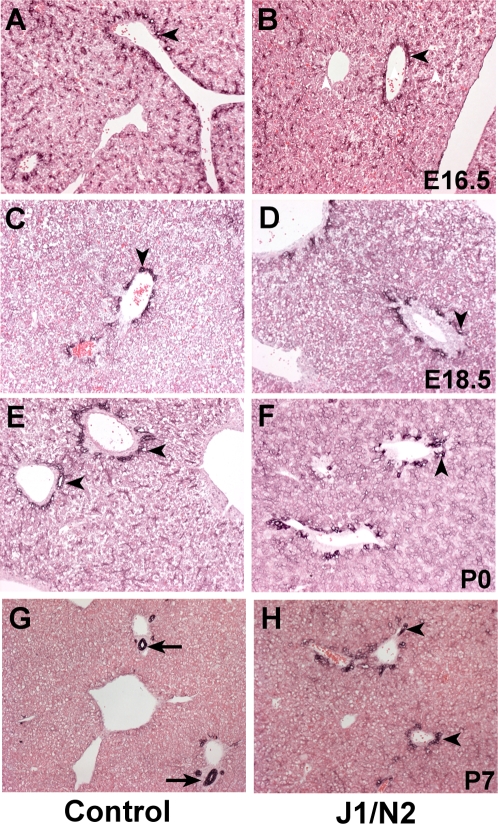
Defects in embryonic bile duct morphogenesis in *Jag1^dDSL^/+ Notch2^del1^/+* mice. Cytokeratin immunostaining of control littermate and *Jag1^dDSL^/+ Notch2^del1^/+* liver sections at the indicated ages. A,B. At E16.5, both control (A) and *Jag1^dDSL^/+ Notch2^del1^/+* (B) have formed a partly bilayered ductal plate (arrowheads). C–F. Over the next several days, focal dilations (arrowheads) form in the ductal plate of both control and mutant embryos. Other regions of the ductal plate begin to regress. G,H. At P7, the focal dilations have formed epithelial bile ducts incorporated into the portal mesenchyme (arrows) in the control liver (G), while the *Jag1^dDSL^/+ Notch2^del1^/+* liver (H) exhibits only ductal plate remnants (arrowheads).

Our previous study [15] analyzed late embryonic and postnatal livers, and did not establish whether bile duct paucity in mice doubly heterozygous for a *Jag1* null allele (*Jag1^dDSL^*) [16] and a *Notch2* hypomorphic allele (*Notch2^del1^*) [19] was due to defects in differentiation of bile duct precursors from the bipotential hepatoblast, or whether it was due to defects in morphogenesis of the ductal plate. Therefore, we analyzed livers of *Jag1^dDSL^/+ Notch2^del1^/+* double heterozygous mice by cytokeratin immunostaining from embryonic day (E) 16.5 through postnatal day (P) 7. At E16.5 in control littermate embryos, cytokeratin immunostaining revealed the presence of a partly bilayered ductal plate at the interface of the portal mesenchyme and the liver parenchyma (Fig. 1A). Over the next several days, the ductal plate remodels by a process in which focal dilations appear between the two cell layers of the plate (Fig. 1C,E). By P7, some of these focal dilations give rise to patent epithelial bile ducts incorporated into the portal mesenchyme (Fig. 1G), while the remainder of the ductal plate involutes. Cytokeratin immunostaining of liver sections from *Jag1^dDSL^/+ Notch2^del1^/+* double heterozygous mice revealed that they were very similar to control littermate sections through at least P0. In the *Jag1^dDSL^/+ Notch2^del1^/+* mice, a ductal plate formed (Fig. 1B) and focal dilations appeared (Fig. 1D,F). However, postnatal remodeling to form a patent epithelial bile duct did not occur. Instead, as we reported in our initial study [15], by P7 only ductal plate remnants remained in most portal tracts (Fig. 1H). These results indicate that in the *Jag1/Notch2* double heterozygote mouse, bile duct paucity results from defects in bile duct morphogenesis, not from defects in differentiation of bile duct precursors from the bipotential hepatoblast.

### Liver-specific *Notch2* deletion results in defects in bile duct morphogenesis, but not ductal plate formation

Our previous study was the first to implicate a critical role for the *Notch2* gene in bile duct formation [15]. The Notch2 protein is expressed in periportal hepatoblasts near or adjacent to Jag1-expressing cells surrounding the portal veins in mice [15], [17], [20]. Further support for a critical role for the *Notch2* gene in bile duct formation and/or maintenance comes from recent studies on Alagille syndrome patients. While improved mutation detection protocols can now identify *JAG1* mutations in approximately 94% of patients diagnosed with Alagille syndrome [21], there are still some Alagille syndrome patients in whom no *JAG1* mutations can be identified. Recently, heterozygous *NOTCH2* mutations were identified in a subset of Alagille syndrome patients who lack *JAG1* mutations [22].

**Figure 2:**
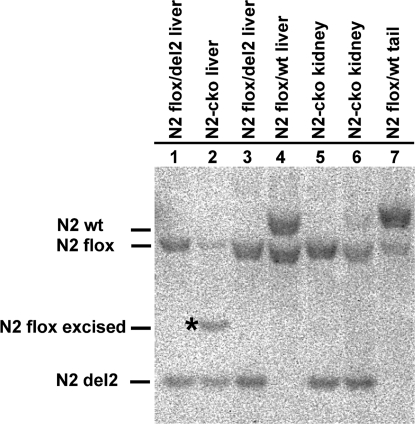
Conditional excision of the *Notch2^flox^* allele by the *Alb1-Cre* transgene. Southern blot of *Sac*I/*Eco*RI- digested DNA isolated at P4. Excision of the *Notch2^flox^* allele (asterisk indicates the excised allele) was observed only in liver DNA of *Notch2-cko* mice (lane 2), but not in kidney DNA of *Notch2-cko* mice (lanes 5,6). Genotype of *Notch2-cko* mice is *Notch2^flox^*/*Notch2^del2^*; *Alb1-Cre*/+.

To specifically assess the role of the *Notch2* gene in bile duct formation in mice, we disrupted *Notch2* function in the liver utilizing mice expressing *Cre* recombinase under the control of the Albumin 1 promoter (*Alb1-Cre*) [23], [24]. We crossed *Notch2^flox^*/*Notch2^flox^* mice with mice doubly heterozygous for the *Alb1-Cre* transgene and either the *Notch2^del2^* or *Notch2^del3^* alleles. Both of these *Notch2* mutant alleles behave genetically as null alleles [25]. Offspring with the genotypes *Alb1-Cre/+*; *Notch2^del2^*/*Notch2^flox^* or *Alb1-Cre/+*; *Notch2^del3^*/*Notch2^flox^* were analyzed. Since no differences were detected in the phenotypes of the *Alb1-Cre/+*; *Notch2^del2^*/*Notch2^flox^* and *Alb1-Cre/+*; *Notch2^del3^*/*Notch2^flox^* mice, mice of both genotypes were designated *Notch2-cko* (for *Notch2* conditional knockout) in this report. Excision of the *Notch2^flox^* allele was observed in liver DNA of *Notch2-cko* mice, but not in kidney DNA of these mice (Fig. 2).

**Figure 3:**
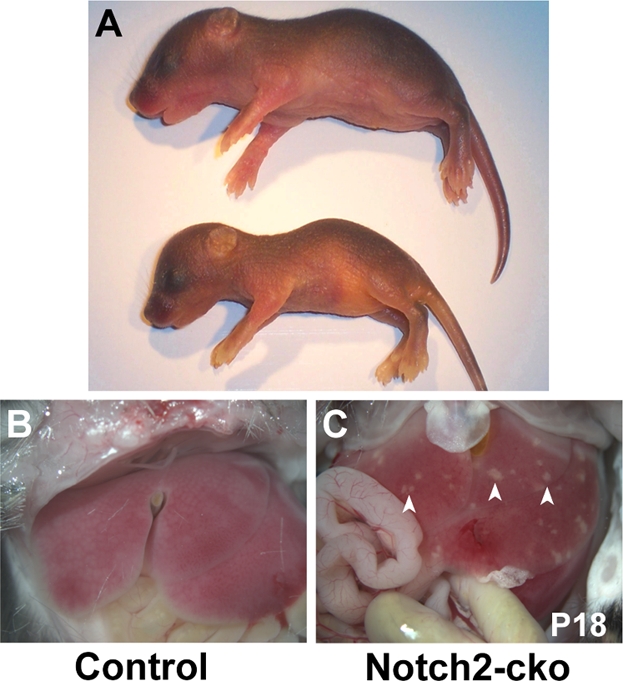
Jaundice and bile duct paucity in *Notch2-cko* mice. A. *Notch2-cko* mouse (bottom) and control littermate (top) at P3. The *Notch2-cko* mouse exhibits jaundice and growth retardation. B,C. Livers at P18. The *Notch2-cko* liver exhibits focal areas of necrosis (arrowheads).

*Notch2-cko* mice were smaller than their littermates (Fig. 3A), and at P8-P9 were approximately 19% lighter than their littermates (4.3±0.1 grams *Notch2-cko* versus 5.3±0.1 grams control littermates). This weight difference was maintained through at least 4–5 weeks of age, when *Notch2-cko* mice were approximately 15% lighter than their littermates. Gross examination of the livers of the *Notch2-cko* mice revealed that *Notch2-cko* livers exhibited focal areas of necrosis (Fig. 3C). These necrotic areas may arise from disruption of bile acid flow, since bile acids are strong detergents and buildup in the liver can lead to necrosis, fibrosis and cirrhosis [6].

**Figure 4:**
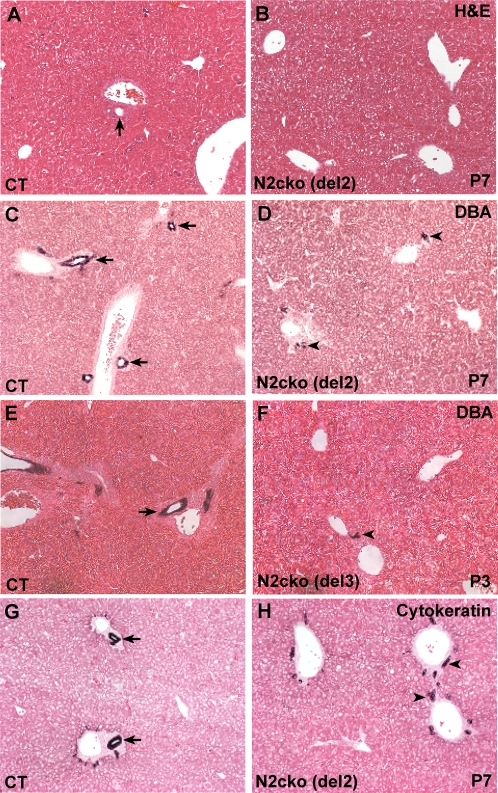
Defects in bile duct formation in *Notch2-cko* mice using either the *Notch2^del2^* or *Notch2^del3^* alleles. A,B. Hematoxylin and eosin-stained sections at P7 of control littermate (CT) and *Notch2-cko* mice using the *Notch2^del2^* allele. Bile ducts (arrow) are observed in the periportal region of the control littermate (A), but not the *Notch2-cko* mouse (B). C–F. DBA lectin staining. C,D. Control littermate and *Notch2-cko* mice using the *Notch2^del2^* allele at P7. E,F. Control littermate and *Notch2-cko* mice using the *Notch2^del3^* allele at P3. DBA-positive cells form patent bile ducts (arrows) adjacent to the portal veins in control mice (C,E). In *Notch2-cko* mice using either the *Notch2^del2^* (D) or *Notch2^del3^* (F) allele, small numbers of DBA-positive cells (arrowheads) are present adjacent to the portal veins, but these cells have not formed patent ducts. G,H. Cytokeratin immunostaining of control littermate and *Notch2-cko* mice (*Notch2^del2^* allele) at P7. The ductal plate of the control liver (G) has remodeled into epithelial bile ducts (arrows), while the *Notch2-cko* liver (H) exhibits only ductal plate remnants (arrowheads).

The biliary tract defects exhibited by *Notch2-cko* mice were very similar to those exhibited by *Jag1^dDSL^/+ Notch2^del1^/+* mice (compare Fig. 1 with Fig. 4). Examination of histological sections of the livers of *Notch2-cko* mice revealed that few morphologically identifiable bile ducts were present (Fig. 4B). Analysis of *Dolichos biflorus* agglutinin (DBA) lectin expression, a cholangiocyte marker [26], revealed that DBA-positive cells formed patent bile ducts adjacent to the portal veins in littermate control mice (Fig. 4C,E). In *Notch2-cko* mice, DBA-positive cells were present in small numbers adjacent to the portal veins, but these cells were not arranged into patent epithelial ducts (Fig. 4D,F). Biliary tract defects were similar using either the *Notch2^del2^* (Fig. 4B,D) or *Notch2^del3^* (Fig. 4F) allele in combination with the *Notch2^flox^* allele. Similarly to portal tracts of *Jag1^dDSL^/+ Notch2^del1^/+* mice, cytokeratin immunostaining revealed that by P7 only ductal plate remnants were detected in most *Notch2-cko* portal tracts (Fig. 4H), while well-formed bile ducts incorporated into the portal mesenchyme were present in the littermate controls (Fig. 4G).

At 4–5 weeks of age, clinical chemistry analysis of serum revealed that, as a group, *Notch2-cko* mice had elevated levels of alkaline phosphatase, alanine aminotransferase, and total bilirubin (Table 1). Elevated levels of these parameters are indicative of liver and biliary dysfunction. However, some *Notch2-cko* mice had alkaline phosphatase, alanine aminotransferase, and total bilirubin levels within the normal range. We also tested blood urea nitrogen levels, which when elevated is indicative of kidney dysfunction. As expected, blood urea nitrogen levels in *Notch2-cko* mice were not elevated (Table 1), in contrast to *Jag1^dDSL^/+ Notch2^del1^/+* mice [15].

**Table 1:** Blood Chemistry Analysis of *Notch2-cko* Mice.

Genotype	*n*	Alkaline Phosphatase	Alanine Aminotransferase	Blood Urea Nitrogen	Total Bilirubin
*Notch2-cko*	23	366±34	135±27	24±0.7	0.71±0.14
Controls	29	246±9	52±9	22±0.8	0.49±0.02

Serum from 4–5 week old *Notch2-cko* mice and their littermates were analyzed for the indicated parameters. Values shown are the mean (in International Units/Liter)±standard error of the mean. All genotypes other than *Notch2-cko* were combined for controls.

### Notch signaling regulates bile duct morphogenesis independently of HNF6 and HNF1β expression

Previous studies have shown that biliary tract morphogenesis is dependent on the transcription factors Hepatocyte Nuclear Factor-6 (*Hnf6*; *Onecut1* – Mouse Genome Informatics) and HNF1β (*Tcf2* – Mouse Genome Informatics). Mice homozygous for a targeted null mutation of the *Hnf6* gene [27], or with liver-specific deletion of the *Hnf1b* gene [28], fail to properly remodel the ductal plate to form patent bile ducts and exhibit persistence of ductal plate remnants. HNF1β expression was strongly downregulated in livers of *Hnf6*-null mice, indicating that the *Hnf6* gene functioned upstream of the *Hnf1b* gene [27].

**Figure 5:**
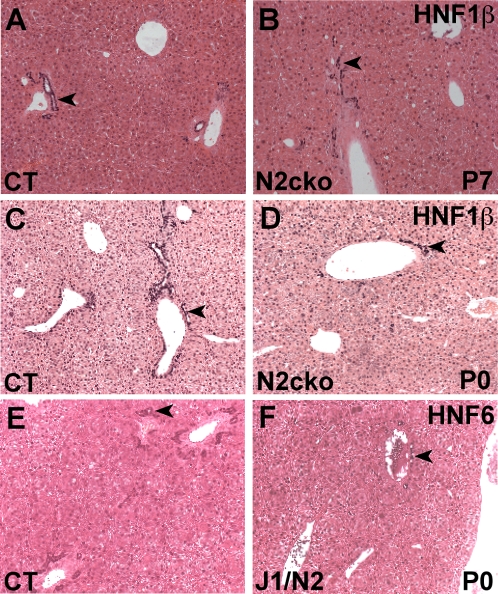
Notch signaling regulates bile duct morphogenesis independently of HNF6 and HNF1β. A–D. HNF1β expression in *Notch2-cko* mice at P7 and P0. E,F. HNF6 expression in *Jag1^dDSL^/+ Notch2^del1^/+* mice at P0. The HNF1β and HNF6 proteins were expressed similarly in the periportal region (arrowheads) of both control littermate and mutant livers.

We tested by immunohistochemistry whether the HNF6 and HNF1β proteins were expressed in the periportal region of *Jag1^dDSL^/+ Notch2^del1^/+* and *Notch2-cko* mice. HNF1β protein expression was observed in the periportal region of *Notch2-cko* mice at P0 and P7 (Fig. 5B,D). Similarly, HNF6 protein expression was observed in the periportal region of *Jag1^dDSL^/+ Notch2^del1^/+* mice at P0 (Fig. 5F). These data suggest that the etiology of the bile duct morphogenesis defects in the Notch pathway mutants is independent of the function of the HNF6 and HNF1β proteins. Independent functioning of the Notch pathway and the HNF6/HNF1β pathway is supported by the finding that *Jag1* and *Hes1* expression is unaffected in fetal livers of *Hnf6*-null mice [27].

## Discussion

The Notch signaling pathway is frequently utilized to specify cell fate during bipotential cell fate decisions [8], [9], so an attractive model to explain the defects in bile duct formation in *Jag1^dDSL^/+ Notch2^del1^/+* mice was reduced differentiation of cholangiocytes from the bipotential hepatoblast. The first indication that this model was likely incorrect came from analysis of mice homozygous for a null mutation of the *Hes1* gene, which encodes a basic helix-loop-helix protein that is a downstream effector of the Notch pathway. The *Hes1*-null mice formed a relatively-normal ductal plate consisting of cytokeratin- and DBA-positive cholangiocyte precursors, suggesting that the primary defect in these mice was not in the initial bipotential cell fate decision of the hepatoblast [17]. However, by P0 in wildtype littermates, patent bile ducts were beginning to form, while none were evident in the *Hes1*-null mice. Unfortunately, *Hes1*-null mice die at birth from severe central nervous system defects [18], precluding the analysis of later stages of ductal plate remodeling and bile duct morphogenesis in these mice.

Our analysis of *Jag1^dDSL^/+ Notch2^del1^/+* mice supports the model that Notch signaling regulates ductal plate remodeling and bile duct morphogenesis rather than cholangiocyte differentiation, and suggests that the *Jag1/Notch2*-mediated signal responsible for bile duct morphogenesis acts, at least in part, by modulating *Hes1* expression. Our analysis of bile duct formation in *Notch2-cko* mice is consistent with this model. While the *Alb1-Cre* transgene does not delete early enough during embryogenesis to study the role of *Notch2* gene function during ductal plate formation [29], the essentially identical biliary tract defects exhibited by the *Jag1^dDSL^/+ Notch2^del1^/+* and *Notch2-cko* mice at late embryonic and postnatal stages strongly suggest that these defects arise by the same mechanism in both mouse models. However, it remains possible that Notch signaling may play some role in cholangiocyte differentiation, since none of the three mouse models analyzed (*Hes1*-null mice, *Jag1^dDSL^/+ Notch2^del1^/+* mice, and *Notch2-cko* mice) are likely to be entirely deficient in Notch signaling when the cholangiocyte-hepatocyte cell fate decision is made.

In contrast to *Notch2* deletion, deletion of the *Jag1* gene in liver hepatoblasts did not lead to defects in bile duct development [30], suggesting that *Jag1* expression in endothelial cells and/or vascular smooth muscle cells was sufficient for signaling to *Notch2*-expressing hepatoblasts during ductal plate remodeling and bile duct morphogenesis. Interestingly, this study also demonstrated that in mice that were compound heterozygotes for a *Jag1* null allele and the *Jag1* conditional allele deleted in hepatoblasts, a subset of animals exhibited bile duct proliferation [30]. Other recent studies support a model in which cholangiocyte differentiation is controlled by a gradient of Activin/TGFβ signaling that is controlled by the expression of Onecut-family transcription factors, such as HNF6 and Onecut2 (OC2) [31], [32]. Our results suggest that Notch signaling regulates bile duct morphogenesis independently of the Activin/TGFβ/Onecut pathway.

In summary, we demonstrate here that similar defects in bile duct formation were observed in both *Jag1^dDSL^/+ Notch2^del1^/+* and *Notch2-cko* mice. However, *Jag1^dDSL^/+ Notch2^del1^/+* mice exhibit defects in many organ systems other than the biliary tract, such as the heart and the kidney [15]. We suggest that liver-specific deletion of the *Notch2* gene in *Notch2-cko* mice represents an improved and more specific model than *Jag1^dDSL^/+ Notch2^del1^/+* mice for studying the role of Notch signaling during bile duct morphogenesis and remodeling.

## Materials and Methods

### Mice

*Jag1^dDSL^*, *Notch2^del1^*, *Notch2^del2^*, *Notch2^del3^*, and *Notch2^flox^* mice were described previously [15], [16], [19], [25]. Albumin-Cre (*Alb1-Cre*) mice [23], [24] were obtained from the Jackson Laboratory. To produce *Alb1-Cre/+*; *Notch2^flox/-^* mice (referred to as *Notch2-cko*, for *Notch2* conditional knockout), *Notch2^flox/flox^* mice were mated to mice heterozygous for both the *Alb1-Cre* transgene and either the *Notch2^del2^* or *Notch2^del3^* alleles. Animal maintenance and experimental procedures were in accordance with the NIH Guidelines for Animal Care and Use and the principles of the Helsinki Declaration, and were approved by the Institutional Animal Care and Use Committee of the Jackson Laboratory.

### Immunohistochemistry and lectin binding

The antibodies and lectins used in these studies were rabbit polyclonal anti-human cytokeratins (Dako, Cat. A0575); rabbit polyclonal anti-HNF1β (Santa Cruz, Cat. sc-22840); rabbit polyclonal anti-HNF6 (Santa Cruz, Cat. sc-13050); and biotinylated *Dolichos biflorus* agglutinin (DBA) lectin (Vector Laboratories, Cat. B-1035). Mutant sections were either *Jag1^dDSL^/+ Notch2^del1^/+* or *Notch2-cko*. Other genotypes, with the exception of *Jag1^dDSL^/+*, were used as littermate controls. No differences were noted in the phenotypes exhibited by the different control genotypes.
